# Endothelial plasticity across PTEN and Hippo pathways: A complex hormetic rheostat modulated by extracellular vesicles

**DOI:** 10.1016/j.tranon.2023.101633

**Published:** 2023-03-09

**Authors:** Elizabeth Orozco-García, D.J. van Meurs, JC. Calderón, Raul Narvaez-Sanchez, M.C. Harmsen

**Affiliations:** a Physiology and biochemistry research group – PHYSIS, Faculty of Medicine, University of Antioquia, Colombia; b Department of Pathology and Medical Biology, University of Groningen, University Medical Center Groningen, Hanzeplein 1 (EA11), Groningen 9713 GZ, The Netherlands

**Keywords:** Vascularization, Angiogenesis, Endothelial cells, miRNAs, lncRNAs, Exosomes, Cancer, Cell communication, EC, endothelial cell, ECM, extracellular matrix, EVs, Extracellular vesicles, HIF-1α, Hypoxia-inducible factor 1 alpha, lncRNA, long noncoding RNAs, miRNA, microRNA, ncRNAs, Non-coding RNAs, PC, pericytes, PI3K, phosphoinositide 3-kinase, PTEN, phosphatase and tensin homolog deleted on chromosome 10, VEGFA, vascular endothelial growth factor 2, vSMC, vascular smooth muscle cells, TAZ, transcription activator with PDZ binding motif, YAP, Yes-associated protein

## Abstract

Vascularization is a multifactorial and spatiotemporally regulated process, essential for cell and tissue survival. Vascular alterations have repercussions on the development and progression of diseases such as cancer, cardiovascular diseases, and diabetes, which are the leading causes of death worldwide. Additionally, vascularization continues to be a challenge for tissue engineering and regenerative medicine. Hence, vascularization is the center of interest for physiology, pathophysiology, and therapeutic processes. Within vascularization, phosphatase and tensin homolog deleted on chromosome 10 (PTEN) and Hippo signaling have pivotal roles in the development and homeostasis of the vascular system. Their suppression is related to several pathologies, including developmental defects and cancer. Non-coding RNAs (ncRNAs) are among the regulators of PTEN and/or Hippo pathways during development and disease. The purpose of this paper is to review and discuss the mechanisms by which exosome-derived ncRNAs modulate endothelial cell plasticity during physiological and pathological angiogenesis, through the regulation of PTEN and Hippo pathways, aiming to establish new perspectives on cellular communication during tumoral and regenerative vascularization.

## Highlights

• Vascularization continues to be a challenge for medicine and tissue engineering.
• Tumor cell-derived exosomes stimulate angiogenesis through PTEN.
• PTEN that differentially controls proliferation to give vessel stability. In non-endothelial cells PTEN blocks the cell cycle but, in VEGF-activated endothelial cells, PTEN releases these from their hyper-mitogenic arrest.
• PTEN and Hippo pathway components converge in the regulation of endothelial patterning during angiogenesis.
• miRNAs and lncRNAs control the "dose" of activation of the PTEN and Hippo pathways, determining the physiological to pathological transition.

## Graphical abstract

**Figure:**
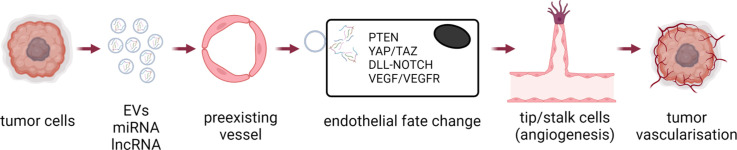

## Introduction

The vasculature is a complex, heterogeneous, and plastic system, which adapts to the tissue requirements to cope with microenvironmental challenges. Blood vessels are prone to respond to variations in mechanical stimuli such as cyclic strain and fluid shear stress that may keep endothelial cells (ECs) in quiescence or induce their activation. Such activation is followed by phenotypic adaptations of ECs and blood vessels which safeguard critical processes such as nutrient supply, waste product disposal, inflammation, and other processes that require cell-to-cell communication. Important vascular responses, namely *vascular adaptation* include (a) angiogenesis, i.e. the formation of new capillaries from existing vessels; (b) changes in vascular reactivity, where the vessel diameter adjusts to specific tissue needs; (c) changes in the architecture of the vascular wall due to reorganization of ECs, pericytes, vascular smooth muscle cells (vSMCs), and fibroblasts as well as the extracellular matrix (ECM) [1].

Cancer is a pathology that drives vascular adaptation, where tumor cells develop strategies to become irrigated in response to the local hypoxic microenvironment. According to these strategies tumors can be classified as pro-angiogenic or non-angiogenic. Angiogenic tumors grow vessels from the pre-existing vasculature toward the tumor site. Non-angiogenic tumors use alternative strategies such as vascular mimicry to meet their metabolic demands to sustain proliferation and metastasis [2]. These alternative strategies vary depending on the tissue of origin, the development status of the tumor, or the anti-tumoral therapy used [3].

The tumor suppressor phosphatase and tensin homolog deleted on chromosome 10 (PTEN) is frequently inactivated in tumor cells [4]. PTEN loss causes constitutive activation of the class I phosphoinositide 3-kinase (PI3K) pathway and is associated with increased endothelial Hypoxia-Inducible Factor 1α (HIF-1α) expression and vascularization stimulation through the PI3K/AKT/VEGF-ET-1 signaling pathway [5]. Furthermore, tumor angiogenesis is enhanced in PTEN-deficient models [6,7]. In contrast, PI3K signaling inactivation by PTEN impairs vascular sprouting by attenuating EC migration and proliferation. PTEN is also linked to vascular homeostasis through PI3K- independent mechanisms.

Tumor development and dissemination depend on cell communication to regulate pivotal processes such as vascularization. Besides the classical signaling through soluble (growth) factors, tumors exploit exosomes to influence their microenvironment. Exosomes are membrane-shelled extracellular vesicles (EVs) that mediate intercellular molecular communication, contributing to a wide range of biological processes in health and disease. Environmental alterations stimulate signaling pathways that influence exosome production rate and content. Tumor cells release around ten-fold more exosomes compared to non-tumoral cells [8], emphasizing the importance of these vesicles for tumor communication. Otherwise, non-coding RNAs (ncRNAs) which include microRNAs (miRNAs) and long non-coding RNAs (lncRNAs), directly affect gene expression profile, and signaling pathway activity and therefore change the cell fate and plasticity.

Exosomal ncRNAs from tumors and ECs regulate the expression of surface receptors, growth factors, ECM components, and signaling molecules involved in vascular adaptation [9]. Some of these ncRNAs influence PTEN signaling during development and pathology, inducing migration, proliferation, and survival in EC and stromal cells. These microenvironmental changes favor the increase in vascular density [10,11]. Moreover, specific exosomal molecule enrichment is associated with the development, progression, and/or outcome of several diseases such as cancer [12]. In this review, we summarize the current knowledge on the mechanisms by which tumor cell-derived exosomal ncRNAs regulate the endothelial phenotype and tumor vascular adaptations *via* PTEN signaling pathway.

## PTEN: a multi-tool protein phosphatase

With an estimated 200,000 different phosphorylation sites in a cellular proteome, the human genome encodes about 612 kinases and 170 phosphatases [13,14]. Interestingly, this small number of phosphatases revert the actions of the dominating sets of kinases, balancing their reduced gene numbers by high protein abundance; suggesting that individual phosphatases have multiple roles and are critical for homeostasis [14].

Many types of allelic losses and sequence alterations in chromosomal region 10q23 in several human cancers led to the search for a tumor suppressor gene in this region, receiving various names including PTEN [6], gene mutated in multiple advanced cancers 1 (*MMAC1*) [15] and transforming growth factor-*β*-regulated and epithelial-cell enriched phosphatase 1 (*TEP1*) [7]. PTEN encodes a ubiquitously expressed lipid and protein phosphatase of 403 amino acids and about 47.1 KDa in humans. The N-terminal domain contains the protein tyrosine phosphatase motif and the C-terminal has a C2 domain which has a membrane phospholipids affinity essential for the lipid phosphatase function [16]. PTEN has different subcellular localizations: membrane, cytoplasm, nucleus, and mitochondria, where its distribution is kept under strict control [17]. PTEN is an evolutionarily conserved and functionally non-redundant protein with crucial roles in vascular development, angiogenesis, motility, proliferation, and metabolism [18]. PTEN is also described as a haploinsufficient tumor suppressor, i.e., one functional allele is not sufficient to sustain a wild-type condition, whereby subtle variations in PTEN dose predispose to tumorigenesis in a tissue-specific manner. Hence, PTEN is on the list of ‘quasi-insufficient’ tumor suppressor genes [19,20]. The homozygous deletion of PTEN is embryonic lethal due to aberrant angiogenesis which underpins its relevance in endothelial biology.

### PTEN regulation

**Fig. 1:**
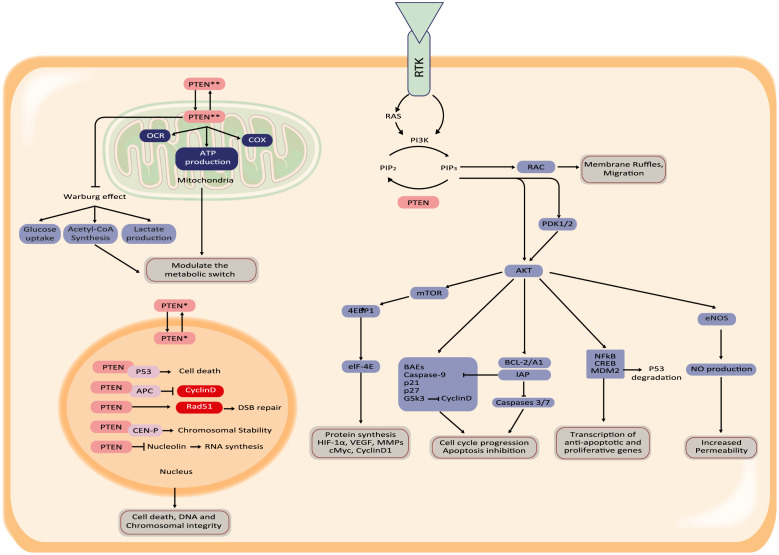
Schematic overview of general PTEN signaling. PTEN is a phosphatase that antagonizes the PI3K/AKT axis, while PTEN also acts as a transcriptional co-factor in the nucleus. In mitochondria, PTEN influences energy metabolism.

PTEN expression is regulated through transcriptional factors, epigenetic silencing (transcriptional); miRNAs silencing (post-transcriptional), and phosphorylation, SUMOylation, ubiquitination, redox regulation, acetylation, S-nitrosylation (post-translational). Its transcription is positively regulated by early growth-regulated transcription factor 1 (EGR1), peroxisome proliferator-activated receptor γ (PPARγ), and P53 among others, having effects on proliferation, cytoskeletal organization, apoptosis evasion, and metabolic adaptations. Additionally, PTEN transcription is negatively regulated by the Nuclear factor kappa-light-chain-enhancer of activated β cells (NFκβ) and Transforming growth factor-beta (TGFβ), as well as by methylation, and miRNAs that target PTEN mRNA. The most widely described role of PTEN is to antagonize the PI3K action through its lipid phosphatase activity at the plasma membrane [15]. PTEN dephosphorylates the lipid substrate phosphatidylinositol [3], [4], [5]-triphosphate (PIP3), converting it back into phosphatidylinositol [4,5]-biphosphate (PIP2), leading to reduced PIP3 levels and signaling. An overview of this process is shown in Fig. 1.

PI3K is a part of the PI3K/AKT/mTOR pathway. Therefore, a negative regulation of PI3K signaling by PTEN inhibits both protein synthesis through mTOR activity and phosphorylation of proteins involved in translational control. PTEN also acts as a negative regulatory signal for the PI3K mitogenic signaling pathway. For in-depth details, the reader is directed to Ref. [21]. During the last decade, other interesting functions for PTEN have been described. Nuclear translocation of PTEN has a role in chromosome stability through the interaction with CEN-P in primary mouse embryonic fibroblasts [22] and is also required for homologous recombination repair of DNA double-strand breaks induced by irradiation in cancer cells (e.g. glioblastoma, colorectal, and breast) [23]. PTEN phosphorylation by ataxia telangiectasia mutated (ATM) kinase in response to DNA damage in cancer cells, is associated with PTEN nuclear translocation and autophagy induction through p-JUN-SESN2/AMPK pathway [24]. Otherwise, PTEN also influences the metabolic switch through the expression of an alternative translation product, PTENα. PTENα stimulates mitochondrial cyclooxygenase (COX) activity through the maintenance of COX hypo-phosphorylation, increases ATP production, and helps to recruit PTEN to the mitochondria [25]. Consistent with the previous, mitochondrial PTEN is linked to increased oxygen-dependent metabolism in normal and tumor hepatic cells. Overexpression of PTEN not only prevents proliferation and migration but also reverts the Warburg effect from glycolysis to oxidative phosphorylation, increasing the oxygen consumption rate [26] (Fig. 1).

The identity of other protein substrates for PTEN is still under study and is also linked to vascular homeostasis through mechanisms independent of PI3K signaling. Growing literature demonstrates that PTEN is versatile beyond its canonical function in the AKT signaling pathway, but PTEN also requires both lipid and protein phosphatase activity for tumor-suppressing functions [27].

## Vascularization during development and pathology

Vascular development is fundamental for multicellular organisms. During tumor growth, tumoral cells expand rapidly and lose access to the bloodstream, generating a vascular homeostasis disruption, leading the tumor to adopt different strategies to increase the capillary density. Three distinct vascularization processes are recognized: (1) angiogenesis, (2) vasculogenesis and (3) arteriogenesis. Tumors primarily depend on angiogenesis which is the hypoxia-driven sprouting of novel vascular branches from pre-existing vessels. This process is often called sprouting or sprouting angiogenesis. In contrast, vasculogenesis is a primarily growth factor-driven process, done by the new formation of a vascular network from aggregating precursor ECs or even adult ECs. Finally, arteriogenesis is the fluid shear stress-induced maturation of pre-existing collateral arteries to functional conduits [28,29].

However, tumors also have specific non-angiogenic vascularization strategies which are not observed in normal tissues, like *vessel co-option* (the tumor grows exploiting pre-existing vessels), *vascular mimicry* (the cancer cells form structures, like channels, lined out by cancer cells), and *cancer stem-like cells differentiation into ECs* [2]. Some tumors are completely non-angiogenic. Also, non-angiogenic clusters of cells can transform into small angiogenic tumor islets, which progress to large vascularized tumors that metastasize, or even angiogenic aggressive tumors can switch to non-angiogenic growth in response to antitumoral therapy [2]. The strategy used by a tumor depends on the stage of development, and physiological and environmental conditions. Besides altering vascularization processes and vascular wall remodeling, tumors also induce changes in vascular reactivity to adapt to the tumor's needs [1,30].

### Sprouting angiogenesis

Sprouting angiogenesis is the most extensively studied vascularization strategy. The angiogenic switch (quiescent vasculature is activated to sprout new capillaries) is triggered by changes in the relative balance of inducers and inhibitors of angiogenesis, while the main inducer is hypoxia [31]. In normoxic conditions, conserved proline residues in HIF-1α are continuously hydroxylated by prolyl hydroxylases, targeting HIF-1α for proteasomal degradation. However, hypoxia deactivates lysyl oxidases and HIF-1α is stabilized, translocated to the nucleus, and activates hypoxia-responsive gene transcription through binding to hypoxia-response elements (HRE) in their promoters. This signaling drives the transcription of pro-angiogenic factors such as vascular endothelial growth factor (VEGF), endothelial nitric oxide synthase (eNOS), endothelin-1 (ET-1), metalloproteinases 2 and 9 (MMP2 and MMP9). The VEGFA/VEGFR2 signaling stimulates PI3K signaling, which activates the serine/threonine kinase AKT. In turn, the AKT family member AKT1 activates eNOS expression to promote NO release. Meanwhile, ET-1 exerts sustained positive feedback on the HIF-1α signaling and its mitogenic and anti-apoptotic effects. The joint effect of these molecules is capillary sprouting through increased vessel permeability, EC proliferation, and migration [32].

In pathological conditions, local hypoxia, oxidative stress, and metabolic derangements drive tumor cells into a pro-angiogenic state, leading to the development of new tumor-penetrating vessels adapted to the tumor. The morphology of the resulting vessels is highly abnormal both structurally and functionally, disorganized, with irregular shapes and high tortuosity [1,33]. Also, due to the lack of pericyte support, tumor vessels are not able to maintain their shape and function, making those vessels leaky to support the exchange of gas, nutrients, and waste products but also to support metastasis. Paradoxically, tumors have a large number of microvessels, but these fail to perfuse properly because of diameter mismatches at vascular branch points [34]. This defective perfusion generates and maintains the metabolic switch, which includes fast energy production and increased carbohydrate fermentation. In consequence, the environmental pH lowers and exacerbates this defective angiogenesis in a positive feedback loop that reinforces the malignant environment [35] Also, disturbances in hemodynamic forces (fluid laminar shear stress, and cyclic strain) derived from under-perfused tissues facilitate the tumor progression through an increase in the cancer-associated fibroblasts [36], [37], [38].

### Endothelial cell patterning during angiogenesis

**Fig. 2:**
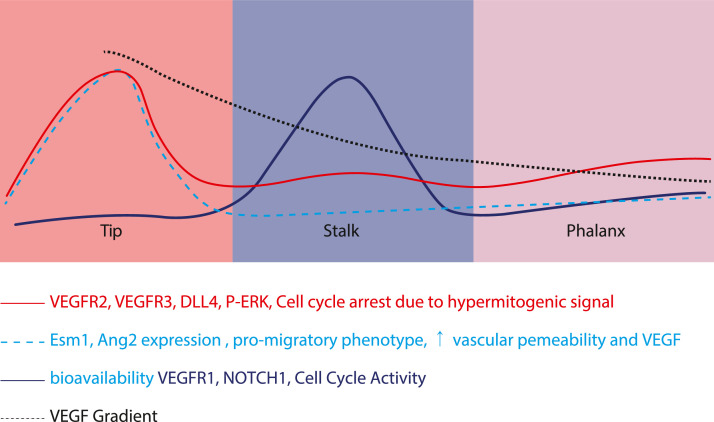
Cellular patterning during angiogenesis. Tip cells are non-proliferative cells that form the leading edge of the protruding vascular tube. Immediately, juxtaposed to the tip cells are the proliferative stalk cells that essentially contribute to the lengthwise extension of the vascular tubes. After proliferation, stalk cells reach quiescence and become mature so-called phalanx cells.

During angiogenesis, ECs form transient but phenotypically specialized EC populations. The *tip cell* EC phenotype is highly motile, non-proliferating, and polarized. This phenotype is associated with high levels of VEGFA/VEGFR2, which induces filopodia formation and extension through Cdc42 signaling (migratory phenotype), which guides to migration towards gradients of VEGFA [39], and helps with vasodilation due to an increase in eNOS expression [40]. The ECs with the s*talk* phenotype are located at the base of the sprout, proliferate, establish adherent/tight junctions, and form the vascular lumen. *Phalanx cells* are lumenized, non-proliferating, and immobile cells (Fig. 2).

Besides EC participation, correct and fully functional vessel development also implies mural cell recruitment. The platelet-derived growth factor B (PDGF-B) is secreted by ECs as a homodimer (PDGF-BB) and mediates the dimerization of its receptor in mural cells. This interaction of PDGF-BB/PDGFR is essential to recruit mural cells such as pericytes or vSMC, which regulate the proper vessel organization and capillary network maturation [41]. *Tip cells* also secrete angiopoietin-2 (Ang-2) and exert an autocrine proliferating signaling over ECs through the interaction of Ang-2 with the receptor Tie2. Recruited pericytes secrete angiopoietin-1 (Ang-1) that outcompetes Ang-2 from their joint receptor Tie-2. However, Ang-1 has no signaling activity, buffering the proliferation consistent with reduced VEGF-A secretion, and causing a complete arrest of EC proliferation. Therefore, a decrease in PDGFR signaling and Ang-1 expression concomitant with an increase in Ang-2/VEGF-A prevents the mural cell integration, supporting continued ECs proliferation and vessel destabilization during the tumor vascularization process [42].

Several molecules like VEGF, PDGF, and Ang-1 exert their action largely through a transitory PI3K/Akt and MAPK activation. A deranged activation of this signaling pathway triggers vascular development defects due to a disrupted specification and recruitment of vascular cells. Constitutively active PI3K/Akt expression *in vivo*, causes fetal vascular malformations and bleeding due to a failure in vascular development, and is associated with enhanced tumor angiogenesis, which suggests that the PI3K/Akt pathway is a key axis in vessel dynamics in ECs [43].

## PTEN in vascularization process: canonical and non-canonical pathways

When considering the molecular mechanisms controlling vascularization through PTEN the most extensively investigated mechanism involves the canonical phosphatase activity of PTEN over PI3K/AKT signaling. PTEN deficiency in ECs causes vascular remodeling defects and contributes to both susceptibility to new tumorigenic mutations and accelerated tumor growth [43]. *Ex vivo* studies have shown that PTEN upregulation inhibits microvessel sprouting in arterial rings and pro-angiogenic processes in ECs including migration, tube formation, reduction in VEGF/ET-1 expression, and signaling. Conversely, PTEN silencing reverses that inhibition and triggers angiogenesis [44].

### PTEN in endothelial patterning during angiogenesis

The specialization in ECs during angiogenesis correlates with increased Delta-like 4 (Dll4) expression in *tip cells*, which binds to and activates Notch 1 in adjacent *stalk cells* [45]. This specialization mechanism is transient and reversible, but indispensable for sufficient regulation of angiogenesis by preventing the *stalk cell* from becoming a new *tip cell* [46,47] (Fig. 2). In *tip cells*, the elevated VEGF/VEGFR2 signaling stimulates high Dll4 expression which blocks Notch signaling in these cells, increases filopodia protrusions, and arrests cell proliferation. Meanwhile, Notch signaling is increased in *stalk cells*, leading to a decrease in VEGF signaling, through reduced expression of VEGFR2/3 and enhanced expression of VEGFR1 and PTEN. Additionally, the number of filopodia is reduced while the cell cycle is increased both *in vitro* and *in vivo* [46], [47], [48]. This mechanism reinforces the *tip cell* position and suppresses the *tip cell* phenotype in *stalk cells*. During this transition, the lagging *stalk cells* adopt a *phalanx cell* phenotype which is a quiescent cell type and the prelude to newly formed quiescent blood vessels (Fig. 2).

It seems contradictory, that the expression of the antiproliferative PTEN is increased in *stalk cells*, because they are widely described to be proliferative, unlike *tip cells*. However, endothelial PTEN (*in vitro* and *in vivo)* is upregulated in *stalk cells* during vessel development through Notch signaling, by interaction with Dll4 on *tip cells* [48]. Why are the cells that have the greatest mitogenic stimulation not proliferating, and the ones that are proliferating express more PTEN? *Tip cells* express high VEGFR2 while producing high VEGF [49], generating a hyperactivation of VEGF/VEGFR2 signaling, which leads to high activation of the downstream MAPK pathway and ERK phosphorylation. This exacerbated mitogenic stimulation induces *tip cells* to exit the cell cycle, in a process known as hypermitogenic arrest, contributing to the induction of *tip cell* features, which include the already mentioned non-proliferation and pro-migratory phenotype [50].

Interestingly, overexpression of Notch signaling in *stalk cells* generates impaired proliferation and a gradual loss of ECs during development. If Notch signaling is lost, the cells do not “win” the vascular growth competition. In consequence, there is a transitory increase in the number and density of ECs without increasing the frequency of proliferating cells. Most likely by an increase in the cell cycle speed followed by a pronounced cell cycle arrest, which in total does not increase the proportion of the proliferating cells and impairs the appropriate vascular progression [50]. Data from this research show that most of the vessels are formed from cells with “normal” Notch levels [50]. Therefore, the physiological Notch activity *in stalk cells* is related to a decrease in hypermitogenic signaling (VEGF/VEGFR1 and MAPK/ERK phosphorylation). This allows the *stalk cells* both, to come out of the hypermitogenic arrest, and slow EC proliferation to prevent a premature expansion and exhaustion of cycling ECs in the angiogenic front [50].

Since the effect of a molecule depends on the context and the dose, PTEN expression in cells with a "normal" mitogenic signal decrease the proliferation. However, Dll4/Notch 1/PTEN signaling in cells under hypermitogenic arrest could help the cells to decrease the hypermitogenic signal and exit from the arrest to start cycling. As *stalk cells* proliferate, they escape from the influence of tip cells (Dll4 signal), Notch signaling wanes, and the balance is gradually tilted and PTEN helps transition from a proliferative to a quiescent phenotype, *phalanx* EC. If PTEN is lost, Notch signaling fails to limit *stalk cell* proliferation and results in defective sprout lengths and patterning [48]. The PTEN loss in ECs from zebrafish resulted in hypervascularization, ectopic vessel formation, and embryonic lethality [51]. Also, PTEN haploinsufficient zebrafish were prone to develop hemangiosarcoma at later life stages. All these vascular alterations were mediated by increased expression of VEGF-A after PTEN loss [51].

Most of the research focuses on *tip* and *stalk cells*, losing sight of the *phalanx cells*. Although PTEN itself is not required for Notch-dependent *tip/stalk* selection, phosphatase-dependent and -independent activities of PTEN are important to regulate *stalk cell* proliferation, vascular density, and vessel growth [48]. Also, most genetic studies are done using models of overexpression or underexpression and assign a pro or anti-angiogenic function to a given genetic product or signaling pathway, while in physiological conditions the dosage can be determinant for the cell plasticity and fate. Considering the above, PTEN could work as a hormetic rheostat helping the cells to reduce the hypermitogenic signal to make them able to cycle but later controlling the proliferation in the molecular transition between *stalk* and *phalanx* cells to give stability to the vessel.

### Dual PTEN and Hippo communication in ECs: a strong feedback loop

Hippo signaling comprises the core kinases MST1/2, SAV1, MOB1, and LATS1/2. MST1/2 interaction with SAV1 activates MST1/2. MST1/2 phosphorylation leads to MOB1 phosphorylation, which in turn phosphorylates LATS1/2. The Hippo signaling is *on* when this cascade is phosphorylated and activated. Subsequently, Yes-associated protein (YAP)/transcription activator with PDZ binding motif (TAZ), effectors of the Hippo signaling, are phosphorylated, and retained in the cytoplasm or degraded. When the Hippo pathway is *off*, YAP/TAZ is not phosphorylated or actively dephosphorylated and shuttled to the nucleus, where it forms a complex with DNA-binding transcription factors including TEAD, Smads, and TBX5. The complex induces the expression of a wide range of genes (e.g., cell junctions, cytoskeletal regulators, kinases, and secreted proteins) which are involved in proliferation, survival, migration, and mechanical sensing [52]. In fact, YAP and TAZ have been identified as key mechanotransducers that detect mechanical stimuli and relay signals to control transcription of downstream genes involved in cellular mechanoresponses. The major upstream mechanosensors for YAP/TAZ signaling are integrins, G protein-coupled receptors, enzyme-linked receptors (e.g. receptor tyrosine kinases), and ion (calcium) channels (e.g. Piezo). These mechanosensors are modulated by disturbed blood flow, stretching or loss of cell contact, changes in cell geometry, or ECM stiffness, whose mechanotransduction elicits downstream transcriptional responses after YAP/TAZ activation or repression. Some of the effects on endothelial cells of disturbed mechanosensing are increased migration and proliferation and increased resistance to apoptosis (reviewed in Ref. [53])

An interesting association between PTEN and Hippo pathway is described in gastric cancer [54]. PTEN inactivation promotes gastric cancer cell proliferation and migration *in vitro*. In PTEN dominant-negative mutant cells, decrease the expression of SAV1, LATS1, LATS2, MOB1, and p-YAP, also decrease the MOB1–LATS1/2 interaction, and increase the expression of YAP/TAZ and YAP is redistributed to the nucleus [54]. A high expression and nuclear localization of YAP are associated with a more aggressive tumor phenotype and a shorter disease-free survival time for gastric cancer patients. In contrast, wild-type PTEN cells show the opposite effect over these Hippo signaling components [54], revealing a connection between those pathways.

**Fig. 3:**
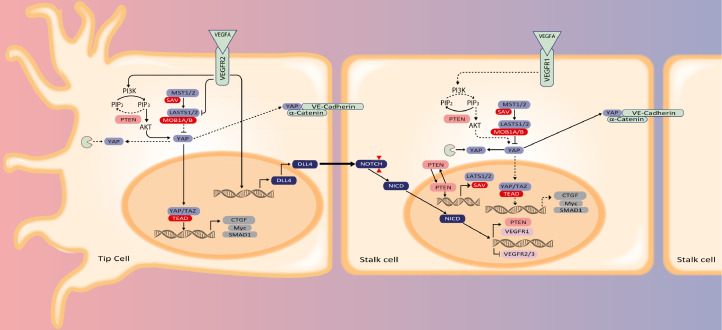
Molecular signaling in angiogenesis. In tip cells, VEGF/VEGFR2 signaling abrogates PTEN and activates AKT which causes nuclear localization of the YAP transcription factor. Nuclear YAP suppresses pro-proliferative signaling. In addition, VEGF signaling suppresses NOTCH expression while upregulating the NOTCH ligand Delta-like 4 (Dll4). The Dll4 activates NOTCH at the surface of stalk cells after which the NICD activates the expression of VEGFR1 while inhibiting VEGFR2 & 3. The decoy receptor VEGFR1 scavenges VEGF which releases PTEN activity and promotes YAP degradation, but part of the YAP is incorporated in adherens junctions and complexed with VE-Cadherin and α-catenin. These actions render stalk cells proliferative.

Like PTEN, Hippo signaling needs to be *off* in tip ECs and *on* in stalk ECs, regulating together ECs proliferation and patterning (Fig. 3) controlling the sprouting angiogenesis and vessel stabilization. Upon stimulation with VEGF, the VEGF/VEGFR2 interaction in brain ECs induce actin cytoskeleton changes and inhibits LATS1/2 and MTS1/2, allowing YAP/TAZ translocation into the nucleus [55]. This link between VEGF and Hippo pathway involves YAP/TAZ DNA binding complexes, controlling the expression of genes related to PI3K/AKT signaling, cell adhesion/ECM-receptor interaction (i.e., *NINJ1, MFAP5*), actin cytoskeleton remodeling (i.e., *MACF1, FLNB, MICAL3, CTGF*), VEGF/VEGFR2 signaling (i.e., *NCK2, SHC2*) and others (Fig. 2) [55]. Deletion of YAP/TAZ in ECs abrogates VEGF signaling; genes that are otherwise upregulated upon VEGF stimulation are decreased after YAP/TAZ depletion [55]. Also, ECs migration, filopodia/lamellipodia formation, and cdc42 regulation are impaired, inhibiting the normal sprouting process. For example, YAP/TAZ-depleted mice have defective sprouting and lumen formation, with extensive multifocal brain hemorrhages and visible leaking areas [55].

With an important role in tumor metastasis and drug resistance, tumor cells can acquire blood vessel-like structures through vascular mimicry. Like physiological angiogenesis, vascular mimicry is regulated by VEGFR. VEGFR-positive breast cancer cells form tubes when treated with VEGF, whereas knockdown of YAP/TAZ inhibits VEGF-induced tube formation. Therefore, the Hippo pathway contributes to VEGF-induced sprouting angiogenesis in ECs, and to vascular mimicry [56].

The exposure of ECs to VEGF inhibits the LATS1/2 kinase activity through stimulation of the VEGFR2-Src kinase complex. During the initiation phase of VEGF-induced endothelial sprouting Hippo kinases are *off*, in other words, expression of LATS1/2 and AMOT is reduced as well as their kinase activity. This causes nuclear translocation of YAP/TAZ and coincides with F-actin polymerization, filopodia extension, and stress fiber formation as the consequence. Thereafter, Hippo kinases turn *on* and YAP/TAZ activities turn *off.* Phosphorylated YAP binds to the 14-3-3 protein and is sequestered by binding to VE-cadherin-binding α-catenin at adherens junctions, followed by enhanced intercellular junction connectivity with the contractile actomyosin network (Fig. 3). Overall, Hippo-YAP/TAZ reads angiogenic signals to promote vascular sprouting and junction maturation [57,58].

Based on the above, the *on*/*off* switch in tip and stalk cells works as follows. In a hypermitogenic arrest context, PTEN is absent (*off*), and PI3K/AKT will be active (*on*), switching *off* the Hippo signaling pathway, and consequently stimulating pro-migratory phenotype in tip cells. Conversely, if PTEN is actively expressed (*on*), PI3K/AKT will be inhibited (*off*), switching *on* the Hippo signaling pathway, and consequently helping the cells to come out of the hypermitogenic arrest, stimulating proliferation and inhibiting the pro-migratory phenotype in stalk cells (Box 1, Table 1, Fig. 3).

**Box 1:**
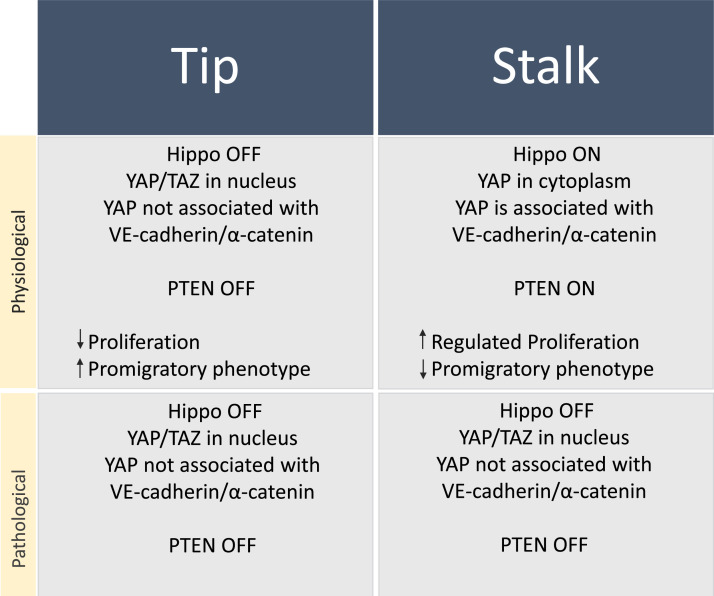
Cytoplasmic and nuclear functions of PTEN and Hippo signaling in EC patterning. Tip and stalk specification can be impaired by persistent silencing by ncRNA, affecting the stability and functionality of the new blood vessel.

**Table 1:** miRNAs targeting PTEN (Ensemble: ENST00000371953) from miRWalk3.0.

miR	Mechanism of action	Cell Type Described	Inside EVs	Hippo Signaling	Ref.
miR-10a/b	Activates pro-metastatic genes, promoting tumor cell invasion and metastasis. Migration.	Ovarian cancer, bladder cancer, breast cancer, esophageal cancer, and gastric cancer cells. ECs.		**+**	[164,165,166,167,168,169,170,171]
^miR-17-5p^*^/3p^	Survival, proliferation, migration, regeneration. EC morphogenesis and angiogenesis. Target TIMP1 Tsp1, PTEN.	ECs, tumor cells		**+**	([78,88,89,172]
^miR-19a/b-1^*^/-2^⁎⁎	Help to suppress the sensitivity to anticancer drugs, and anti-apoptotic. EC morphogenesis and tumor angiogenesis. Regeneration Target TIMP1 Tsp1, PTEN.	Gastric cancer cell line, ECs, leukemia cells (K562).		**+**	[78,88,89,140,143,172,173]
^miR-20a^*^/b-5p/3p^⁎⁎	EC morphogenesis and tumor angiogenesis. Regeneration Target TIMP1 Tsp1, PTEN.	Breast cancer, myeloma cells, ECs.		?	([139,140]
^miR-21^	Proliferation, EMT, angiogenesis. Repair spinal cord microvasculature. Maintaining of vSMC phenotype.	In vitro in vivo models (several tumors, vascular repair, healthy).		+	[83,86,97,134]
^miR-23a/b-3p^	Proliferation, tumor progression, metastasis.	Prostate cancer.			[90]
miR-25-3p***	Up-regulation of miR-25 was correlated migration and invasion.	Esophageal squamous cell carcinoma.			[174]
miR-26a-1-5p/3p	c-Myc increases miR-26a expression. miR-26a activates Akt signaling through direct PTEN targeting and increases VEGF, MMP2 expression. Increases proliferation and invasiveness.	High-grade glioma, glioblastoma multiforme, lung cancer cells		**+**	[90,91,175,176]
miR-26b	Enhance growth, survival, and tube formation through PTEN targeting. Inhibit the proliferation, migration, and EMT in epithelial cells through prostanoid pathway inhibition.	EC, epithelial cells			[177,178,179]
^miR-29a-5p^	Pro-angiogenic YAP induces miR-29 to inhibit PTEN translation which activates PI3K–mTOR signaling to regulate proliferation and cell size.	Lung cancer cells, ECs, epithelial cells, mice.		**+**	^(^[138]^,^[180]
^miR-92a-1-5p^*^/a-2^⁎⁎ ^miR-92b-3p^	EC morphogenesis and tumor angiogenesis. Regeneration Target TIMP1 Tsp1, PTEN.	ECs, leukemia cells (K562).		**+**	[78,89,90,181,182,183]
^miR-93-5p^⁎⁎⁎	High proliferation, anti-apoptotic, and influence tumor size. Cisplatin resistance, suppressing PTEN and over activating AKT pathway. Angiogenesis	Osteosarcoma cells, ovarian cancer, ECs, mouse hearts		**+**	^(^[135,184,185]
miR-103a-3p	Downregulation of angiogenesis inhibitors expression, like TIMP3. Promotes cell transformation, and disrupt the endothelium integrity, increasing the vascular permeability.	Endometrial and hepatocarcinoma cells, ECs.		**+**	[127,128,129,130,131,186]
miR-105-5p	Vascular permeability disruption. Downregulates ZO-1 expression, altering the vascular junctions.	In vitro, ex vivo and in vivo assay in rodents.			[133]
miR-106a-5p/3p⁎⁎/ miR-106b-5p/3p⁎⁎⁎					
miR-130a/b	Proliferation, migration, and anti-apoptotic	Glioma cell lines, squamous cell carcinoma.			[187,188]
miR-181b/c-5p					
^miR-193/a-3p^	Cell proliferation, migration, 5-FU chemoresistance, and in vivo tumorigenicity through PTEN targeting. Contradictory, functions like tumor suppressor miRNA, inhibiting migration, invasion, and EMT in vitro and metastasis in vivo in lung cancer.	Gastric cancer cell lines			^(^[189,190]
^miR-200c-5p/3p^	Alters wound-healing capacity and colony formation. MT1-MMP (Membrane type-1 matrix metalloproteinase) and PTEN expression are regulated.	Pancreatic cancer cell.			
^miR-205-5p^	Directly targets SMAD4, CYR61, CTFG, PTEN. Promotes proliferation, anti-apoptotic, migration, chemoresistance, and tumorigenesis in vivo. Regulated Erb/HER, PTEN/AKT axis. Biomarker for cervical cancer patients.	Ovarian cancers (OC) with poor diagnostic, cervical cancer cells, breast, lung, and NSCLC cancers.			[93,94,95,191,192,193,194]
miR-221-3p	Downregulates PTEN and activates PI3K/Akt signaling. Induce cell survival, proliferation, carmustine, and cisplatin resistance.	Osteosarcoma cells, Epithelial ovarian cancer (EOC) cells			[195]^,^[196]
miR-223	Downregulates PTEN and activates PI3K/Akt signaling.	Ovarian cancer cells			[124]
miR-301a/b	M2 macrophage polarization through PTEN targeting. Promote migration, invasion, EMT, and facilitate the lung metastasis of pericytes.	Pancreatic cancer cells		**+**	[126]
miR-382-5p	Target PTEN and consequent activation of AKT/mTOR signaling. Increases proliferation, migration, and apoptosis avoidance. Increases vascular EC proliferation, migration, and tube formation.	Hemangioma-derived endothelial cell, hypoxia in human gastric cancer cells			[92,197]
miR-425	MALAT1 interacts with miR-425 preventing PTEN silencing. Induce apoptosis	Plasma and plasma exosomes			[141]
miR-494-5p/3p	Target PTEN and subsequent activation of AKT/eNOS pathway. Enhances proliferation, migration, invasion, and promotes angiogenesis.	Hypoxic lung cancer cells, glioblastoma cells, ECs.			[198,199]
miR-638	Dual PTEN and p53-targeting. Cell migration, invasion, proliferation, and anchorage-independent growth.	Exogenous expression model in prostate cancer cells			[163]
^miR-939^				^?^	^(^[132]

⁎ ^miR-17/92 cluster: located in chromosome 13, encodes miR-17, miR-18a, miR-19a, miR-20a, miR-19b-1 and miR-92a-1, lso known as oncomiR-1.^

⁎⁎ ^miR-106a/363 cluster: located on chromosome X, encodes miR-106a, miR-18b, miR-19b-2, miR-20b, miR-92a-2, and miR-363.^

⁎⁎⁎ ^miR-106b/25 cluster: located in chromosome 7, encodes miR-106b, miR-93, and miR-25.^

### Other functions of PTEN in vascular cells

Vascular homeostasis implies not only the ECs but also pericytes and vSMC phenotype and function. Other important downstream targets of the canonical PTEN pathway, are also linked with pathological vascular adaptations. PTEN depletion in mice induces a sustained PI3-kinase-Akt-mTOR signaling, leading to a decrease in vSMCs markers expression including αSMA and calponin. This PTEN loss concomitant with PI3-kinase-Akt enhancing is also linked to NFκβ activation and production of chemoattractant and profibrotic factors like MCP-1, IL-6, and KC/CXCL1, promoting injury-induced vascular adaptation. Interestingly, the PTEN loss was also associated with proliferation and neointima formation [59], promoting a general upregulation of proinflammatory and profibrotic genes [60]. The chemokine stromal cell-derived factor-1α (SDF-1α) is a PTEN downstream mediator. PTEN loss in vSMCs induces SDF-1α expression, and this helps to develop an inflammatory phenotype characterized by the recruitment of bone marrow-derived progenitor cells. The SDF-1α expression also induces vSMC hyperplasia by an autocrine growth loop through the interaction between SDF-1α and its receptor CXCR4. Interestingly, PTEN loss in vSMCs is directly associated with an increase in both HIF-1α expression and nuclear localization in an Akt-dependent manner irrespective of hypoxia [61,62]. Increased expression of PTEN confers protection against damage induced by angiotensin II, decreasing profibrotic and proinflammatory markers [63]. Derived from this, it has recently been identified that the use of 5-azacytidine, an inhibitor of DNMT1 (DNA methyltransferase-1), restores the expression of PTEN, promoting the maintenance of SMC differentiation and reducing pathological vascular remodeling [64]. All the above highlights PTEN signaling to play a central role in the vascular adaptation processes during development and tumor progression, which involve not only the ECs but all the cells that shape the architecture of the vascular wall.

## Non-coding RNAs

The miRNAs and lncRNAs are in the large group of non-coding RNAs [65]. The miRNAs are small RNAs between 18 and 25 nucleotides in length and represent the most widely studied group of small ncRNAs [66]. miRNAs are predicted to target over 60% of all 3′ UTR mRNAs of human protein-coding genes [66]. They mediate their repressive effect through mRNA degradation or translational repression. Also, miRNAs may function as ligands directly binding to receptors, triggering downstream signaling pathways, regulating a plethora of physiological cell processes like differentiation, proliferation, metabolism, angiogenesis, apoptosis, and immune response. For in-depth details on miRNA biogenesis and mechanisms, the reader is directed to Ref. [67]. Besides, lncRNAs are usually larger than 200 nucleotides, which would originate from <1% of the human genome [65], and can also act as post-transcriptional regulators, suppressing the effect of the miRNAs. For in-depth details in lncRNA biogenesis, the reader is directed to Ref. [68].

Like virtually all mRNAs, PTEN mRNA is a target of this miRNAs/lncRNAs expression regulation. Since tumor development demands signaling, metabolic and microenvironmental shifts [69], a dysregulation of homeostatic ncRNA expression is involved in the pathophysiology of cancer [70,71]. The differential ncRNAs expression profiles in healthy and tumor tissues allow ncRNAs to be used as diagnostic and prognostic biomarkers, and for disease therapy design [71,72].

### Angiogenesis and EC phenotype regulation by miRNA *via* PTEN

One of the most widely investigated onco-miRNAs (miRNAs associated with cancer) is miR-21, a multi-pathway regulation miRNA that contributes strongly to cancer pathology [73]. Different oncogenes like Ras trigger the expression of miR-21, stimulating proliferation and neoplastic transformation through the silencing of different tumor suppressor genes [74]. Moreover, the downregulation of tumor suppressor miRNAs may also stimulate tumorigenic processes due to the lack of inhibition of several oncogenes. The miRNA Let-7 is frequently downregulated during the onset and progression of cancer [75]. Let-7 inhibits the expression of Ras and c-Myc, thus, downregulation of miR-Let7 causes overexpression of Ras and c-Myc oncogenes [76,77]. This overexpression of Ras induces overexpression of miR-21, and c-Myc also stimulates a pro-tumor phenotype characterized by increased proliferation, migration, invasion, apoptosis inhibition, and immune escape [78]. These pathological positive feedback loops orchestrate a stimulatory symphony for tumor development such as vascular adaptation.

PTEN targeting by miR-21 in human hepatic, lung, and cervical cancer cells promotes epithelial-to-mesenchymal transition (EMT), angiogenesis and a pro-tumor phenotype [79], [80], [81], [82]. PTEN/PI3K/AKT, TGFβ1, and ERK/Bcl2 signaling are the main signaling pathways through which miR-21 operates in different tumor and vascular cells [83], [84], [85]. In addition, miR-21 also supports angiogenesis by targeting the angiogenesis inhibitor tissue inhibitor of metalloproteinase-3 (TIMP3) and stimulation of MMP2/9 expression and secretion in ECs. This pro-angiogenic role of miR-21 is a determining factor for the repair of the spinal cord microvasculature after injury [86]. Even, the deletion of miR-21 impairs vascularization and promotes apoptosis after ischemia, since it cannot suppress the activation of the PTEN/PI3K/AKT axis [87].

Besides, c-Myc overexpression induces the onco-miRNA miR-17/92 cluster [78]. Most of the members of the phylogenetically conserved cluster miR-17/92 and its two paralogues miR-106a/363 and miR-106b/25, target PTEN. Overexpression of miR-17/92 cluster in cervical and colorectal cancers increases PI3K/AKT/mTOR signaling targeting PTEN expression consequently, inducing pro-tumor phenotype [88]. Expression of the miR-17/92 cluster augments the angiogenic switch mediated by growth factors in a positive feedback loop [89]. VEGF-induced angiogenesis increases the expression of the miR-17/92 cluster, promoting an angiogenic phenotype through PTEN downregulation [89], but also directly repressing the anti-angiogenic factors thrombospondin-1 (TSP-1) and connective tissue growth factor (CTGF), stimulating angiogenesis [78]. Also, miR-23b and miR-26a help to impair PTEN/PI3/AKT signaling, increasing prostate and lung cancer cell proliferation, tumor progression, and metastatic potential [90,91].

Under hypoxia, stabilization of HIF-1α upregulates miR-382 expression. miR-382 targets PTEN leading to PI3K/AKT/mTOR signaling activation and VEGF secretion, promoting angiogenesis in ECs and chorioallantoic membrane [92]. PTEN is also targeted by miR-205, which increases PI3K/AKT/mTOR signaling pathway promoting migration, metastasis, and chemoresistance in lung and cervical tumor cells [93,94]. miR-205 directly targets PTEN, promoting endothelial progenitor cell (EPC) angiogenesis through an increase in MMP‐2 expression *via* AKT/autophagy pathway, helping to deep vein thrombosis recanalization process [95].

Otherwise, nuclear functions of PTEN are necessary to maintain the identity of other cells in the vascular unit like vSMCs [96]. Nuclear localization and interaction of PTEN with the transcription factor Serum Response Factor (SRF) is essential for SRF binding to vSMC promoters to maintain the vSMC differentiation program. SRF negatively regulates the expression of the transcription factor Fos-related antigen 1 (FRA-1) through an increase in the expression of miR-143, which targets FRA-1. FRA-1 in turn mediates the negative regulation of miR-21 promoter activity and this repression of miR-21 promoter activity avoids the translational repression of PTEN [97]. Silencing of SRF also inhibits PTEN expression through a miRNA-dependent mechanism. Loss of the SRF/PTEN axis promotes the vSMCs reprogramming, decreasing the expression of contractile genes and mediating a proliferative and inflammatory vSMC phenotype [96,97]. All the above makes PTEN an essential regulator to maintain the vSMC contractile gene expression. The loss of PTEN in intimal vSMCs is associated with the stabilization of atherosclerotic lesions and restenosis [60]. Thus, hinting at vSMC PTEN participation in the stabilization of a neo-vasculature in ischemic tissue injury and tumor progression. In this regard, the PTEN targeting by several miRNAs impacts vascular cells and EC phenotype during both development and malignant phenotype promotion. Given all the above, what is the PTEN right dose for proper vascularization? Not too much, nor too little. This will depend on the context: development, therapeutic or pathological.

## Exosomal biogenesis and biological role in cell-to-cell communication

First described by Harding and co-workers [98] and Pan and Johnstone [99], exosomes are EVs produced through the secretory pathway and released by exocytosis by many cells. Exosomes comprise membranous nanoparticles that range between 30 and 150 nm in diameter. These can carry a repertoire of bioactive molecules such as proteins, lipids, carbohydrates, and nucleic acids i.e., coding- and non-coding RNAs [100].

Inside the cells, early endosomes fuse with endocytic vesicles to incorporate their content and be sorted for recycling, degradation, or exocytosis. During maturation, the transition from early to late endosomes, these vesicles undergo membrane invagination and fission events, generating multivesicular bodies (MVBs) [100]. These MVBs comprise numerous membrane-limited intraluminal vesicles (ILVs). Late endosomes are targeted to either fuse with lysosomes for cargo degradation or fuse with the plasma membrane to release the ILVs to the extracellular environment, after release ILVs are called exosomes [101]. The components of the endosomal sorting complex required for transport (ESCRT) and several Rab proteins (Rab3, −4, −5, −11, and Rab27) are required for MVB and ILV biogenesis. ESCRTs are assembled into four complexes (resp. ESCRT-0, -I, -II, and -III) with associated proteins (VPS4, VTA1, ALIX), which recognize and sequester proteins to the endosomal membrane [102], participate in membrane deformation into buds with sequestered cargo (I, II), and drive vesicle scission, which requires TSG101 and ALIX [103]. CD63 and ALIX have a pivotal role in vesicle sorting [104]. Finally, the dissociation and recycling of the ESCRT machinery requires the AAA-ATPase Vps4 [105]. The biogenesis processes of the extracellular vesicles are reviewed in detail elsewhere [106].

The exosome composition is also influenced by specific biogenesis pathways stimulation, which differs between normal cells and their tumorigenic derivatives [102]. The cells can have ESCRT-dependent and -independent exocytosis. Rab27a is linked to both ESCRT-dependent exocytosis and highly tumorigenic and metastatic exosome production [107]. The miRNAs and proteins inside these exosomes help to coordinate the metastatic cascade [108,109]. Therefore, exosomes facilitate the crosstalk between cells, mediating the reprogramming, transformation, and/or recruitment of several cells such as adipose-derived stem cells to tumor sites [110,111], changing the tumor microenvironment to promote angiogenesis [112], and metastasis [113].

The tumor microenvironment also influences the molecular exosome profile. Stressors like hypoxia, pro-inflammatory and metabolic challenges, activate transcription factors that change the cell expression profile and in consequence the exosomal content [109]. Exosomes from glioblastoma multiforme (GBM) patients and hypoxic GBM cells accumulate high concentrations of pro-angiogenic mediators like MMP9, pentraxin 3, IL8, PDGF-AB/AA, CD26 (aka dipeptidyl-peptidase-4), and plasminogen activator inhibitor 1 (PAI-1), stimulating proliferation, migration, and angiogenesis in ECs, mouse aorta rings and human GBM xenografts [114]. Interestingly, tumor cells exert an effect on surrounding ECs, while ECs also influence tumor cells through EV communication. Hence, constant crosstalk among cells persists. Cadherins mediate isotypic cell homophilic interactions, ECs express VE-cadherin, and epithelial breast cancer cells can be positive or negative for E-cadherin depending on the malignity. However, VE-cadherin expression is induced in breast cancer cells when they are co-cultivated with ECs [115]. As a “trojan horse”, the tumor cells can move through the endothelium having an EV-inducible homophilic interaction, maintaining the integrity of the endothelial monolayer, helping to both process tumor invasion and vascular mimicry [115].

Like other molecules, PTEN can also be regulated through exosomal communication [113]. Mouse embryonic fibroblasts secrete PTEN in exosomes into the extracellular environment conserving their phosphatase activity in the recipient cells having effects on the AKT pathway and decreasing cell proliferation [116]. All of the above reinforces the exosomes as potent mediators of intercellular communication, actively participatory in the preparation of the extracellular microenvironment supporting pivotal processes during cell specification, development, repair, and disease.

### Exosomal PTEN-targeting miRNAs in endothelial phenotype and tumor angiogenesis

The exchange of vesicle-loaded ncRNAs redirects the cell phenotype but also the neighboring cellular environment. Several cell types ranging from MSCs (therapeutic context) to tumor cells (pathological context) influence angiogenesis-PTEN-related mechanisms in paracrine ways. That paracrine intercellular communication is modulated through exosomes-derived ncRNAs, including miRNAs and lncRNAs, helping to redirect the target cell functions alike a cuckoo laying its eggs in the nest of a host bird. Table 1 lists the main miRNA whose target is PTEN in several tissues. The regulation by miRNAs is highly versatile, not only do specific mRNAs harbor binding sites for multiple miRNAs but a single miRNA generally binds to multiple target mRNAs transcribed from genes that often reside in the same signaling pathway. Thus, one miRNA might hit an entire pathway.

#### miR-21, miR-205-5p, miR-223 and miR-301

miRNAs play key roles in vascular and tumor development, targeting molecules like VEGF/VEGFR family members and PTEN signaling. In prostate tumor cells, miR-21-5p targets PTEN which activates HIF-1α. This upregulates VEGF expression and secretion which promotes tumor angiogenesis [83]. The exosomal miR-21 leads to STAT3 activation, which increases VEGF levels in recipient cells, leading to angiogenesis and malignant transformation of human bronchial epithelial cells [117]. Exosomal miR-21 from hepatocellular carcinoma cells targets PTEN mRNA in proximal hepatic stellate cells (HSC), activating and transforming these HSCs into cancer-associated fibroblasts (CAFs). CAFs secrete pro-angiogenic factors such as VEGF promoting tumor vascularization and remodeling the ECM to generate a pro-tumorigenic microenvironment [118]. miR-21 secreted on exosomes from colorectal cancer cells also promotes angiogenesis and vascular permeability [119]. miR-205-5p influences development and cancer by regulating cell proliferation, migration, tissue polarity, and morphogenesis [120]. Like a double-edged sword, pathological loss of miR-205 creates discontinuities in the basal membrane of prostate glands by an expression reduction in both laminin-332 and the cellular matrix receptor integrin α6β4, which favors malignant transformation [121]. Also, a pathological increase of miR-205-5p in different cancer cells and tumor-derived exosomes inhibit the PTEN expression causing the upregulation of PI3K/AKT signaling, promoting proliferation, and angiogenesis [95].

In a multi-pathway communication mechanism, the local hypoxia, the metabolic switch induction, and in consequence the low pH, change the cellular miRNA expression profile in tumor cells and ECs alike [122]. This altered expression profile confers to the cells the ability to modify the microenvironment through different mechanisms including exosomal communication. Altered exosomal lipid/protein/miRNAs expression profiles influence both exosome release and uptake, increasing the exosome fusion with cell membranes [123]. Hypoxic epithelial ovarian cancer cells recruit macrophages and stimulate these to produce exosomes enriched in miR-223, which target the PTEN-PI3K/AKT pathway, helping the cancer cells to proliferate and evade chemotherapy [124]. M2-type macrophages are anti-inflammatory and immunosuppressive and secrete IL-10, TGFβ, and VEGF, leading to proliferation, tissue repair, and angiogenesis [125]. Exosomes from hypoxic pancreatic cancer cells harbor miR-301a-3p that induces M2 polarization through PTEN expression inhibition, and consequently activation of PI3K/AKT/mTOR signaling. This promotes migration, invasion, and EMT, facilitating lung metastases [126].

#### miR-103, miR-105, and miR-939

Several miRNAs described to target PTEN also target adherens and tight junction proteins in ECs, like VE-cadherin and zonula occludens 1 (ZO-1), respectively. The loss of these junction proteins constitutes a determinant step in vasculogenesis during development, but also in physiological and pathological sprouting angiogenesis. miR-103 targets PTEN, with a context-dependent outcome. miR-103 targets PTEN [127] and TIMP-3 [128] in endometrial cancer cells, stimulating growth, invasion, and apoptosis decrease. Upregulation of miR‐103 was observed in oxidized-LDL-treated human ECs, a model for atherosclerosis. Direct PTEN targeting by miR-103 allows MAPK signaling activation to promote the expression of inflammatory cytokines (MMP‐9, MMP‐1, IL‐6, IL‐1β) and endoplasmic reticulum stress markers (GRP78, CHOP, XBP‐1, and GRP94); and miR-103 inhibition attenuates these markers through PTEN expression recovery [129]. Additionally, EVs derived from hypoxic lung cancer cells decreased macrophage PTEN levels caused by EV-miR-103a transfer, increasing the activation of AKT and STAT3 as well as the expression of several immunosuppressive and pro-angiogenic genes related to M2 phenotype [130]. Interestingly, xenografts of miR-103 overexpressing-hepatocellular carcinoma cells (HCCs) in mice, help tumor metastasis and alter the EC phenotype *via* exosomal communication. The exosomal miR-103-HCC xenograft derived, destroys the ECs adherent junctions integrity by direct targeting of VE-cadherin, p120‐catenin, and ZO-1. This facilitates the transendothelial invasion of tumor cells, increasing the vascular permeability, the number of tumor cells in blood circulation, and the hepatic and pulmonary metastases, compared to control mice [131]. miR-939 is upregulated in human breast cancer cells, particularly in the most aggressive subtypes. Indeed, lymph node-positive tumors overexpressing miR-939 have an increased risk of relapse as compared with those with lower levels of miR-939 and lacking lymph node involvement. miR-939 is expressed and released in exosomes of triple-negative breast cancer cell lines and its uptake in HUVECs downregulates the junctional protein VE-cadherin [132]. Exosomal transference of miR-105 downregulates the tight junction protein ZO-1 in ECs [133]. Together, miR-939 and miR-105 increase vascular permeability and destroy EC integrity, leading to enhanced metastasis.

### Exosomal YAP/TAZ-targeting miRNAs in endothelial phenotype and tumor angiogenesis

As discussed above, a strong feedback loop between PTEN and Hippo signaling pathway drives vascular adaptations in ECs during development and disease (Fig. 3). This signaling network may seem to act as an on/off switch, counteracting the balance of signaling in *tip* and *stalk*. This allows for angiogenic stimulation and warrants the proper maturation of the newly sprouted vessels to render these to function as microvessels. Any perturbance of this communication i.e., disturbance of the balance in tip and stalk cells, has effects on regeneration and recovery of tissues after damage. Moreover, it may cause the formation of non-functional vessels.

Interestingly multiple endogenous miRNAs redundantly converge on PTEN and Hippo signaling pathways to activate robust and sustained proliferation and EC patterning (Table 1). For example, the miR-17/92 cluster, which targets PTEN, also induces cardiomyocyte proliferation and regeneration through modulation of Hippo signaling through LATS2 targeting, thus increasing YAP nuclear translocation and transcriptional activity (Table 1). Also, miR-21 induce proliferation and angiogenesis through Hippo interference in cancer cells [134]. In ECs and ischemic hearts, overexpression of miR-93 was directly associated with a decrease in LATS2 expression and an increase in nuclear YAP, in other words, inactivation of the Hippo pathway. This protective response prevented ischemia/reperfusion damage to the endothelium by promoting angiogenesis (cardiac and *in vitro*), increasing microvascular density in the infarcted myocardium, and decreasing endothelial activation (ICAM-1 and VCAM-1 staining) [135].

### Exosomal lncRNA in endothelial phenotype and tumor angiogenesis *via* PTEN

Like miRNAs, lncRNAs are post-transcriptional regulators. One particularly interesting way of lncRNA functioning is the endogenous sponge function, in which the lncRNA binds a target molecule e.g. a miRNA, and thereby prevents this molecule from binding to its original target [136]. In this way, the lncRNA abolishes the potentially inhibitory effect of its targeted miRNA on specific mRNAs. Such lncRNAs that regulate the expression of other transcripts by competing for miRNAs are referred to as competing endogenous RNAs (ceRNAs) [137]. In this last chapter, we focus on lncRNAs that regulate PTEN signaling.

Growth arrest-specific 5 (GAS5) is a lncRNA that functions as a ceRNA for the PTEN target miR-29-3p. Lung cancer-derived exosome profiles showed reduced expression of GAS5 compared to the exosomes of healthy lung cells. Low GAS5 levels promoted tumor angiogenesis, while high levels of GAS5 resulted in inhibited proliferation and tube formation, and increased apoptosis [138]. Interestingly, the tumor increases angiogenesis by suppressing the amount of GAS5 in its exosomes. This suggests that there is an important role for healthy lung cells in maintaining homeostasis in the lung through the exosomal delivery of GAS5 to suppress pro-carcinogenic processes. These results show that lung cancer cells regulate tumor angiogenesis *via* PTEN by controlling GAS5 levels in their exosomes (Table 2).

**Table 2:** lncRNAs targeting PTEN.

LncRNA	Endogenous Sponge	Main function	Cell Type	Role exosomes	Interaction PTEN	Effect on angiogenesis	Refs.
PTENP1	Yes	Binding miR-20a and thereby promoting PTEN	Cancer cell	?	Promoting	Suppressed malignant behaviors like metastasis	[139,141]
APC-1	No	Disrupting mRNA Rab5b stability and thereby reducing exosome production/release	Cancer cell	APC-1 affects the production/release?	No direct effect, possibly indirect by affecting exosome production	Suppressing	[149]
GAS5	Yes	Binding miR-20a-3p and thereby promoting PTEN	Endothelial cell	Transport GAS5 from the cancer cells into the ECs	Promoting	Suppressing	[138]
H19	Yes	Promotes angiogenesis in the tumor microenvironments and related with vascular repair	Endothelial cell	Transport H19 from the cancer cells into the ECs	Downregulation	Promoting	[[142], [143], [144],^200^]
POU3F3	No?	Promotes angiogenesis after the exosomal transfer from the glioma cell into the endothelial cell. Downregulate GAS5	Endothelial cell	Transport POU3F3 from the cancer cells into the ECs	?	Promoting	[147,201]
MALAT1	Yes	Therapeutic angiogenesis and metastasis	Coronary vessels	Transport MALAT1 which sponges miR-92a	?	Promoting	[145,146,202]

PTEN pseudogene 1 (PTENP1) is a lncRNA, a member of the PTENP1/miR-20a/PTEN axis. This axis is involved in the progression of breast cancer by mediating cell proliferation, metastasis, and apoptosis [139]. MiR-20a is known as a suppressor of apoptosis and as a promoter of cell proliferation due to binding and thereby suppressing PTEN. PTENP1 is called a pseudogene because it is, just like PTEN, a target for miR-20a. This ceRNA function of PTENP1 promotes PTEN signaling by decreasing miR-20a activity. Consequently, low PTENP1 expression promoted the malignant behavior of breast cells, while overexpression of PTENP1 suppressed breast cancer progression [140]. Furthermore, PTENP1 is also reported as a member of the PTENP1/miR-17/PTEN axis. PTENP1 was demonstrated to be transported from normal cells to bladder cancer cells by exosomes, where it induced a tumor-suppressing effect. In contrast, low levels of PTENP1 showed a malignant effect in these cells [141]. Again, this seems a way in which an exosomal lncRNA is involved in an effort of the body to defend itself against cancer (Table 2).

H19 is a lncRNA that was upregulated in cancer stem cell CD90+ liver cancer cell-derived exosomes, but not in normal liver cancer cell-derived exosomes. These exosomes induced the promotion of angiogenesis in ECs, by increasing VEGF and VEGFR. Besides, H19 overexpression in ECs displayed the same effect [142]. Although H19-mediated interaction with PTEN-targeting miRNAs, such as miR-19a-3p [143] and miR-675 [144], has been documented in previous years, the precise pathways in which cancer cell-derived exosomal H19 induces angiogenesis remains to be elucidated. Another lncRNA, metastasis-associated lung adenocarcinoma transcript 1 (MALAT1), acts as an angiogenesis promoter after exosomal delivery to ECs, suppressing miR-92a. Being also a predictor of poor prognosis for epithelial ovarian cancer patients [145,146]. POU3F3 lncRNA also promotes angiogenesis after their transference to ECs by cancer-derived exosomes [147,148] (Table 2). Angiogenesis stimulation *via* downregulation of PTEN after cancer-derived exosomal delivery of H19 and MALAT1 has not yet been reported. However, available literature suggests that these lncRNAs interact in multiple ways with this process, which renders these interesting for future research.

LncRNA-APC1, named after its activator, is a lncRNA active in the APC/PPARα/lncRNA-APC1/Rab5b axis. In colorectal cancer cells, lncRNA-APC1 binds to Rab5b mRNA, leading to reduced stability of this mRNA and decreased RAB5B protein levels. This led to decreased exosome production, inhibition of the overreaction of the MAPK pathway, and suppression of angiogenesis. Low levels of lncRNA-APC1 are associated with metastasis and poor prognosis in colorectal cancer patients [149] (Table 2). Besides the lncRNAs transported in the exosomes, it is important to evaluate other effects of lncRNAs on exosome delivery. Effects of lncRNAs on exosome production, release, and uptake can also contribute to the effect that a parent cell establishes in a recipient cell due to the delivery of its exosomes.

LncRNAs are a relatively novel field in biomedicine that warrants more thorough exploration. Revealing how lncRNAs manipulate signaling pathways can provide a new important element in the understanding of cellular functioning. Especially the interaction with lncRNAs and miRNAs seems to add an extra layer to the interplay of molecules involved in post-transcriptional signaling. In addition to the oncogenic and tumor suppressor miRNAs, mapping of pro-angiogenic and anti-angiogenic lncRNAs can serve as important new information to get a more complete view of the functioning of both PTEN-dependent and PTEN-independent intracellular signaling pathways.

Although not the focus of this review, ncRNAs comprise a class of degradation-resistant RNA, so-called circular RNAs (circRNAs), which have an average half-life of approximately five times longer than mRNAs [150]. Indeed, EVs also carry circRNAs [151]. Whereby, it has been suggested to use circRNA as biomarkers of progression and severity in different diseases, including colorectal cancer [152,153]. In 2017 exoRBase was created, which is a repository of exosome-derived long RNAs (exLRs) derived from RNA-seq data analyses in different human body fluids. The exLRs contain mRNA, lncRNA, and circRNA [154]. Several circRNAs participate in the regulation of PTEN and Hippo signaling, such as circPTEN1 and circ-PPP1R12A, respectively [153,155]. However, the role of cirRNAs in vascular plasticity regulation, beyond tumor angiogenesis models [156], should be the subject of future study.

## Concluding remarks and perspectives: vascular adaptation lessons from cancer cells

A strong feedback loop between PTEN and Hippo signaling pathway seems to rule vascular adaptations (angiogenesis, vascular reactivity, and remodeling of the vessel wall) during development and disease. PTEN has a pivotal role in vascular homeostasis, and its influence lies not only over PI3K signaling pathway. PTEN signaling regulation is a fertile area, as more than 2000 miRNAs are predicted to target PTEN [157]. Although several miRNAs targeting PTEN have been described, there is still a huge gap between the predicted and biologically validated ncRNAs, and a large percentage of validated ncRNAs has been described only in a single context. Some of the ncRNAs that have been validated in different biological models show a context-dependent outcome, which increases the complexity when drawing conclusions with these molecules (Tables 1 and 2).

As a further point of consideration, the miRNA signatures of 2D cultured human ECs massively differ from 3D cultured ECs [158]. miRNA profiling comparing freshly isolated and cultured ECs, revealed major alterations in miRNA signatures between tissue-derived, cultured and aging ECs, differing around 30–40% in miRNA expression in cultured ECs compared to the fresh ECs [158]. Also, the stiffness of the substrates where the cells are planted has an impact on the EC miRNA expression profile and cell fate [159], as mentioned in the part 4.2 of the present review. This research reveals the existence of a mechanosensitive miRNA-based program conformed by 122 microRNA families that target 73 mRNAs encoding cytoskeletal, contractile, adhesive, and ECM, being YAP/TAZ signaling one of the main regulatory targets [159]. Although PTEN is not widely mentioned in that research, this is of special relevance since many of the miRNAs described to regulate this mechanosensitive program can also target PTEN. This indicates that the miRNA profile is highly adaptable and influenceable by the culture conditions, and cultured cells may not always represent the best model system for studying miRNA function, proper *in vivo* validation will remain as the gold standard.

Some of the studies cited here, described endogenous downregulation of PTEN expression by miRNAs. However, we do not know if they also influence exogenous expression. This allows us to determine if the predicted miRNA response elements (MREs) in PTEN are *bona fide* MREs and if the observed miRNA effect on endogenous PTEN is not indirect. Similarly, abundant literature describes the biological phenomena in which a miRNA interferes, but there is a gap in the description of the molecular mechanisms through which they execute its action.

The role of exosomes in pathophysiology as well as in normal physiology is investigated comprehensively during the last decades, with exponential growth in the number of publications associated with exosomes and ncRNAs, especially miRNA. Is well-recognized that ncRNAs packaged in exosomes alter physiological and pathological vascularization, reprogram the recipient cells, and thus change the cellular microenvironment, being PTEN one of the targets. Conversely, the cells’ microenvironment continuously demands the adaptation of the biological cargo in exosomes. Nevertheless, exosomes not only transport miRNAs but a wide variety of molecules with biological activity as lncRNAs. The stoichiometry of exosomal-miRNA transference (relation between copies number of miRNA/exosome/cell) describes that there is no more than one copy of each miRNA per exosome, delineating functional boundaries of exosome-mediated communication, their mechanism, and physiologic relevance over gene expression in a biological context [160,161]. Therefore, trying to explain the effect of exosomes from a single molecule is a reductionistic act. In the future, it will be necessary to integrate expression profiles of the total content of the vesicle, to understand cellular communication in detail, and the entire message.

As Calabrese and Mattson say: “the hormesis is not only constrained by plasticity but also describes the key characteristics of biological plasticity” [162]. Understanding the importance of the dose-response, makes us think about the molecules not only as *on/off* switches but rather as a hormetic rheostat. Transformed cancer cells are characterized by many chromosomal alterations, which include copy number amplifications and loss of heterozygosity in several loci. These genetic alterations imply drastic increases or decreases in the genic product expression (mRNAs/ncRNAs) encoded in these areas [163], changing the proper gene dose expression. In angiogenic tumors, the angiogenic switch must be induced frequently. However, the vascularization of the tumor is highly disordered. Therefore, the analysis of angiogenic factors regulated by tumors is relevant in the mapping of the effector's hierarchy, as well as their implied pathways. Since it is currently easier to inhibit angiogenesis than to induce the formation of functional vessels, this information is important not only to learn how to suppress such angiogenic flux in growing tumors but also to make a bridge to define optimal therapeutic miRNA doses to improve regenerative processes.

## Ethics approval, consent to participate, and consent for publication

Not applicable.

## Funding

This project was supported by the University Medical Center Groningen, (UMCG), the University of Antioquia (Medellin. Colombia), and the Colombian Ministry of Science and Technology (Minciencias) Project 111577757581.

## CRediT authorship contribution statement

**Elizabeth Orozco-García:** Conceptualization, Visualization, Data curation, Investigation, Formal analysis, Writing – original draft, Supervision. **D.J. van Meurs:** Investigation, Formal analysis, Writing – original draft, Visualization, Supervision. **JC. Calderón:** Writing – review & editing, Visualization, Supervision. **Raul Narvaez-Sanchez:** Conceptualization, Visualization, Supervision, Writing – original draft, Writing – review & editing. **M.C. Harmsen:** Conceptualization, Investigation, Formal analysis, Writing – original draft, Writing – review & editing, Visualization, Supervision.

## Declaration of Competing Interest

The authors declare no conflict of interest. The funders had no role in the design of the study, in the collection, analyses, interpretation of data, or in the writing of the manuscript.
